# Rewiring β_2_-adrenergic receptor signaling: harnessing non-canonical GRK functions to treat metabolic diseases

**DOI:** 10.1038/s41392-025-02407-4

**Published:** 2025-09-12

**Authors:** Sean A. Cullum, Andreas Bock

**Affiliations:** 1 Institute of Pharmacology, University Medical Center of the Johannes Gutenberg-University Mainz, Mainz, Germany; 2 Research Center for Immunotherapy (FZI), University Medical Center of the Johannes Gutenberg-University Mainz, Mainz, Germany

**Keywords:** Target identification, Target validation

In a study published recently in *Cell*, Motso et al.^1^ developed a series of G protein-coupled receptor kinase 2 (GRK2)-biased agonists for the β_2_-adrenergic receptor (β_2_AR) which showed efficacy in stimulating muscular glucose uptake without eliciting cardiac side effects typically associated with systemically applied β-agonists.^2^ The candidate drug, compound 15, was well-tolerated in a phase 1 clinical trial and could provide a promising alternative treatment option for type 2 diabetes and obesity.^1^

Type 2 diabetes and obesity represent major and rising global health challenges. In recent years, novel drugs such as GLP-1 agonists (liraglutide, semaglutide) have revolutionized the treatment of type 2 diabetes and obesity. However, these drugs have adverse effects and their long-term efficacy is unclear, highlighting the need for improved therapeutics for metabolic disorders.

The β_2_AR belongs to the superfamily of G protein-coupled receptors (GPCRs) which form the largest class of cell membrane receptors in humans and constitute pivotal drug targets.^3^ Canonically, β_2_AR activation stimulates cAMP production *via* the G_s_ protein. Activated β_2_ARs recruit GRK2 which phosphorylates the receptor’s C-terminus, promoting β-arrestin-recruitment. Subsequently, β_2_ARs internalize but can reinitiate a second signaling wave from subcellular compartments with location-specific functions^4^ (Fig. 1a). Due to its bronchodilatory effects, local activation of pulmonary β_2_ARs by β-agonists represents a key treatment of asthma and chronic obstructive pulmonary disease. The β_2_AR also promotes glucose uptake in skeletal muscle.^2^ However, targeting this receptor to treat diabetes or obesity has so far been hindered by severe cardiovascular adverse effects such as cardiac hypertrophy and increased heart rate and contractility, which have been attributed to β_2_AR-mediated cAMP signaling.^2^

**Fig. 1:**
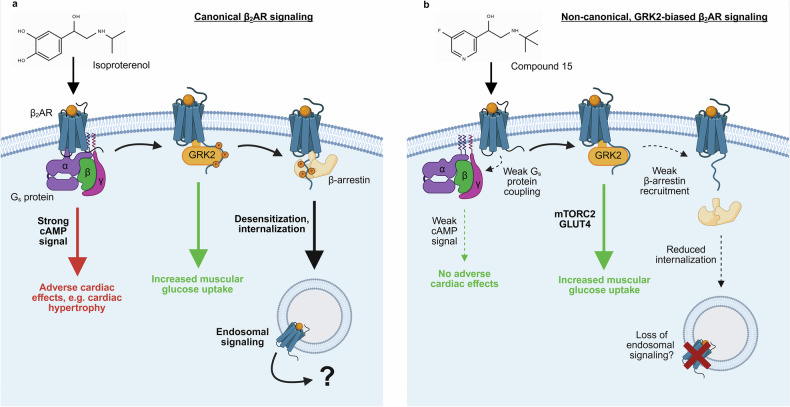
Model of canonical and non-canonical β_2_AR signaling pathways. **a** Isoprenaline-mediated canonical β_2_AR-G_s_ coupling stimulates cAMP production which is associated with adverse cardiac effects such as cardiac hypertrophy. GRK2 phosphorylation of the receptor C-terminus and subsequent β-arrestin recruitment result in β_2_AR desensitization and internalization, where further signaling from subcellular compartments can occur. **b** Compound 15 preferentially promotes a unique β_2_AR complex with GRK2 over G_s_ protein or β-arrestin without phosphorylating the receptor C-terminus. This induces a non-canonical GRK2 signaling pathway which increases muscular glucose uptake without evoking cAMP-associated side effects or β_2_AR internalization. The figure was created using Biorender.com

Motso et al. reasoned that novel β_2_AR agonists that efficaciously stimulate glucose uptake while only weakly activating the G_s_/cAMP/PKA signaling axis could serve as potential drug candidates to treat type 2 diabetes and obesity. Using a combination of virtual similarity-based pharmacophore screening, lead optimization and phenotypic screening, the authors identified several β_2_AR agonists which strongly promote glucose uptake but only partially increase cAMP production. A selection of these agonists (compounds 15, 21, 26) were tested in in vivo experiments in rodents designed to examine both efficacy in regulating glucose metabolism and potential to cause cardiac side effects. Specifically, in diet-induced obesity (DIO) mice, compounds 15, 21 and 26 enhanced glucose tolerance, while compound 15 also reduced fat mass and insulin resistance.^1^ However, in contrast to salbutamol and clenbuterol, these novel compounds caused no increase in DIO mouse heart weight, evoked no or only a minimal increase in contractility of human atria and did not result in myocardial lesions in rats.^1^

Noteworthy, monotherapy with compound 15, or in combination with either liraglutide (GLP-1R agonist) or empagliflozin (SGLT2 inhibitor), elicited greater beneficial effects on both fasting glucose levels and glucose tolerance than either liraglutide or empagliflozin alone, suggesting compound 15 could represent an improvement on existing therapies for type 2 diabetes. Finally, co-therapy of compound 15 with liraglutide prevented muscle atrophy in DIO mice observed with the incretin mimetic alone, indicating the potential of co-administration of these drugs in obesity treatment. Compound 15 was advanced to a phase 1 safety clinical trial, which yielded positive results for tolerability and pharmacokinetics, thus advancing compound 15 to phase 2 efficacy trials.^1^

## What is the molecular mechanism for the striking functional effects of the new β_2_AR agonists in vitro and in vivo?

Using bioluminescence resonance energy transfer (BRET)-based assays, the authors profiled 15 transducer proteins to assess whether the novel β_2_AR agonists preferentially promote engagement of selected transducers relative to isoproterenol, a standard β_2_AR agonist. Interestingly, they discovered that compounds which strongly favored glucose uptake over cAMP production displayed a marked preference for GRK2-recruitment over other transducers like G_s_ protein and β-arrestin2,^1^ revealing a so-called “GRK2 signaling bias”. It is important to note that the novel β_2_AR agonists favor GRK2-coupling but β-arrestin-recruitment and subsequent internalization is strongly impaired, despite the well-established canonical function of GRKs to promote these processes. This striking feature is in line with the fact that β_2_AR activation by compound 15, in stark contrast to isoproterenol, did not result in GRK2-mediated phosphorylation of the receptor’s C-terminus. Together these data suggest that compound 15 and its congeners stabilize a unique β_2_AR/GRK2 complex which does not activate the enzymatic activity of GRK2 toward the receptor but drives glucose uptake in skeletal muscle. This argues for a distinct, non-canonical signaling function of GRK2 (Fig. 1b). Although it is known that GRKs phosphorylate other proteins than GPCRs and also modulate protein function in a phosphorylation-independent manner,^5^ the functional role of non-canonical GRK features has remained enigmatic.

To unravel the signaling functions of such non-canonical β_2_AR/GRK complexes, the authors performed phosphoproteomics from microdissected primary muscle bundles (endogenously expressing β_2_ARs) that were stimulated with compound 15. Relative to vehicle, compound 15 treatment resulted in activation of mammalian target of rapamycin (mTOR), a central component of mTOR complex 2 (mTORC2) (a known regulator of glucose metabolism). Although it remains unclear from this study whether compound 15-stimulated phosphorylation of proteins is mediated by cAMP-dependent protein kinase (PKA), GRK2 or other kinases, the essential roles of GRK2 and mTORC2 in driving compound 15-stimulated glucose uptake were validated by gene knockdown and pharmacological inhibition, respectively.^1^ Lastly, compound 15 promoted translocation of the glucose transporter GLUT4 to myoblast plasma membranes, elucidating the mechanism of glucose uptake by GRK2-biased β_2_AR agonists (Fig. 1b).

## How does this study advance the field?

Two important aspects of this study could have a significant impact on the GPCR field. First, from a therapeutical point of view, GRK2-biased β_2_AR agonists may represent a promising, orally available alternative to current type 2 diabetes and obesity treatments, although it should be noted that evidence of clinical efficacy in patients has not yet been demonstrated. In addition, cardiovascular phenotyping of the drug candidate including its potential effects on cardiac metabolism and blood vessels will be necessary to fully understand the drug’s pharmacological profile. Second, from a conceptual viewpoint, this study broadens our understanding of GPCR biased signaling.^3^ To date, most studies investigating biased signaling have focused on agonists which can preferentially activate G protein-signaling over β-arrestin-recruitment (and vice versa) or ligands which discriminate between different G protein subtypes. The present study demonstrates that GPCR ligands are capable of stabilizing unique GPCR conformational states that preferentially recruit GRK over both G proteins and β-arrestin. Most importantly, these GPCR/GRK complexes appear to have unique signaling functions and are devoid of canonical kinase activity. On another note, the lack of receptor internalization upon stimulation with the biased agonists likely restricts β_2_AR signaling from subcellular compartments (Fig. 1b), thus altering the spatiotemporal signaling landscape, the consequences of which are currently unclear but may also contribute toward the beneficial pharmacological profile of GRK2-biased agonists.

In conclusion, the present study advances the field by establishing that we should consider GRKs not only as intermediate enzymes which desensitize GPCRs through phosphorylation and β-arrestin-recruitment, but also as signaling proteins themselves.
